# Sinisan ameliorates early-life stress-induced depressive-like behaviors by repairing DRN synaptic damage through CaSR

**DOI:** 10.3389/fphar.2025.1508037

**Published:** 2025-05-21

**Authors:** Qingying Yu, Huan Li, Xulan Cui, Liuchang Zhou, Zedan Xie, Shanshan Wang, Di Deng, Jinlan Zhao, Peng Sun, Yafei Shi, Rong Zhang

**Affiliations:** ^1^ Guangdong Provincial Key Laboratory of Translational Cancer Research of Chinese Medicines, Joint International Research Laboratory of Translational Cancer Research of Chinese Medicines, International Institute for Translational Chinese Medicine, School of Pharmaceutical Sciences, Guangzhou University of Chinese Medicine, Guangzhou, Guangdong, China; ^2^ Guangdong Provincial Key Laboratory of Clinical Research on Traditional Chinese Medicine Syndrome, The Second Affiliated Hospital of Guangzhou University of Chinese Medicine, Guangzhou, Guangdong, China; ^3^ Innovation Research Institute of Chinese Medicine, Shandong University of Traditional Chinese Medicine, Jinan, Shandong, China; ^4^ School of Fundamental Medical Science, Guangzhou University of Chinese Medicine, Guangzhou, Guangdong, China

**Keywords:** SNS, depression, dorsal raphe nucleus, calcium-sensitive receptors, synaptic structure

## Abstract

**Indroduction:**

Early-life stress (ELS) is a well-established risk factor for adolescent depression, yet the underlying neurobiological mechanisms remain incompletely understood. The dorsal raphe nucleus (DRN), a key serotonergic center, demonstrates stress-induced synaptic impairments that may underlie depressive phenotypes. Sinisan (SNS), a classical Chinese herbal formula, shows clinical efficacy against mood disorders, but its effects on adolescent stress-induced DRN synaptic damage are unknown.

**Methods:**

Using a maternal separation plus chronic unpredictable mild stress (MSCUMS) model in adolescent rats, we integrated behavioral tests with various neurobiological analyses. Depressive-like behaviors were evaluated, synaptic ultrastructure in the DRN was examined via electron microscopy, and CaSR expression was measured. The therapeutic effects of SNS and the mechanistic role of CaSR were investigated through pharmacological activation (GdCl3).

**Results:**

MS-CUMS induced: (1) depressive-like behaviors, (2) DRN synaptic ultrastructural damage, and (3) Calcium-sensing receptor (CaSR) downregulation. SNS treatment normalized depression/anxiety behaviors, restored CaSR expression and ameliorated synaptic damage. CaSR activation (GdCl3) reversed these deficits, confirming its mechanistic role.

**Discussion:**

These results demonstrate that CaSR mediates ELS-induced DRN synaptic impairment, and SNS exerts rapid antidepressant effects via CaSR upregulation.

## Graphical Abstract

**Figure:**
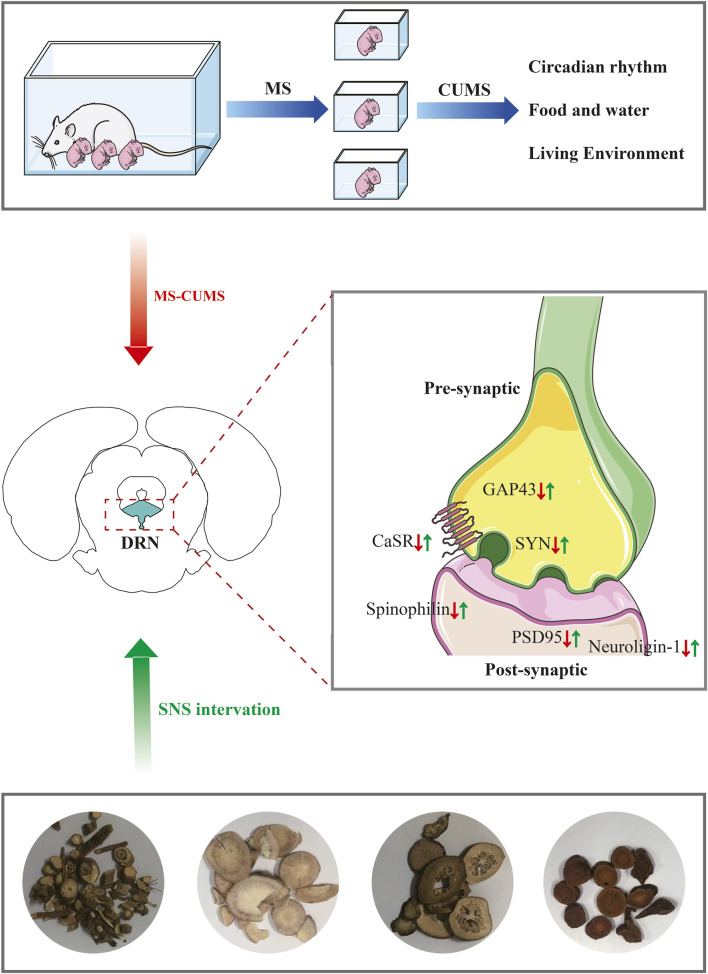

## 1 Introduction

Depression is one of the most common affective disorders in adolescence and is the leading cause of illness and disability among adolescents aged 10–19 years. One study indicated that children exposed to adverse stress had a high risk of developing emotional and psychiatric disorders such as anxiety disorders and depression (Ménard et al., 2016; Saleh et al., 2017), Stress can also induce neuroplasticity and synaptic plasticity (Deng et al., 2022) and results in changes in central neural networks, including prefrontal cortex and hippocampus (Cao et al., 2019). In addition, altered synaptic plasticity and reduced function of neurons in the dorsal nucleus of the middle suture have been reported in the pathophysiology of depression (Fernandez et al., 2016; Yohn et al., 2017). Therefore, ameliorating or even reversing depression-induced impairments in synaptic structure and function represents a critical focus of current research.

Adolescent depression is a psychological condition that occurs in adolescents, which is closely related to the release of 5-hydroxytryptamine (5-HT) synthesis in the brain. Neurons in dorsal raphe nucleus (DRN) are main site for central 5-HT synthesis and secretion, and can project towards almost all brain regions to form the central 5-HTergic nervous system, and thus also become a key site of action for intervening in 5-HT (Paquelet et al., 2022). Recent studies have shown that early life stress significantly alters gene expression in the DRN, which increases the risk of developing mental illnesses such as depression (Bravo et al., 2014). Synaptic plasticity in DRN is a principal factor in the aetiology of depression. Chronic restraint stress has been reported to significantly impair synaptic strength and plasticity in the DRN (Haj-Dahmane and Shen, 2014). Improving synaptic damage and reversing the expression levels of synapse-related proteins alleviated depressive and anxiety-like behaviors (Deng et al., 2022), suggesting that there is a close relationship between depression-like behaviors and synaptic plasticity in DRN.

Calcium-sensing receptor (CaSR), which are widespread at synapses, play important roles in synaptic plasticity and neural signaling (Yamamura et al., 2012). CaSR is involved in the process of synaptic activity by promoting axonal growth and dendritic branching development (Ruat and Traiffort, 2013; Loretz et al., 2009), and mediate neuronal cell growth and differentiation (Vizard et al., 2008; Chattopadhyay et al., 2007; Giudice et al., 2019), which in turn affects transmitter release and regulates learning and memory (Guo et al., 2012). It has been shown that during periods of stress, synaptic plasticity is impaired and the level of CaSR expression in the brain is significantly reduced (Shen et al., 2020). Notably, development and early life are important for synaptic plasticity and neural network construction (Wu et al., 2022). Considering that CaSR expression increases during the growth and development stage and the expression level gradually decreases during adulthood, it can be postulated that CaSR plays a role in the process of brain development (Zheng et al., 2011). Nevertheless, the effect of CaSR in early life stress-induced alterations in synaptic structure and function remains unknown.

Chinese medicine has a history of more than a thousand years of experience in the treatment of depression, so exploring effective interventions for the treatment of depression through Chinese medicine is a new direction for research on the treatment of depression (Chen et al., 2019). This formula Sinisan (SNS) is often used clinically to treat depression (Zhou et al., 2018) because of its effectiveness in treating depression of the stagnation of liver-qi type. SNS is derived from the Shanghan Lun and consists of four botanical drugs, namely, *Bupleurum chinense* DC. (Chai-hu), *Paeonia lactiflora* Pall. (Bai-shao), *Citrus × aurantium* L. (Zhi-shi) and *Glycyrrhiza uralensis* Fisch. ex DC. (Gan-cao), which have the effect of dispersing stagnated liver qi for relieving qi stagnation (Yang et al., 2017). The group’s previous studies have shown that SNS has a good therapeutic effect on animal models of stress-induced depression-like behaviour in early life (Deng et al., 2022). And SNS treatment normalised CaSR protein expression levels in stressed rats brain, indicating the important role of CaSR in SNS to improve depression (Shen et al., 2020). However, the mechanism by which SNS exerts its antidepressant effects via CaSR remains poorly understood. Targeting synaptic structure and function in the DRN may provide a scientific rationale for treating depression, as synapses in brain regions supporting executive, social, and affective functions appear particularly susceptible to modification and responsive to adolescent environmental changes. Therefore, further research is necessary to explore potential mechanisms by which SNS improves synaptic damage of DRN during adolescent depression.

Our study hypothesizes that SNS ameliorates early-life stress-induced behavioral abnormalities in adolescents by modulating CaSR expression and synaptic structure in the DRN.

## 2 Materials and methods

### 2.1 Preparation of SNS

SNS was composed of Chai-hu (NO: 220101), Bai-shao (NO: 220701), Zhi-shi (NO: 220601), and Gan-cao (NO: 220401) in a ratio of 1:1:1:1 from Guangzhou Zhixin Chinese Medicine Beverage Co. (China). The botanical drugs were precisely weighed at a 1:1:1:1 ratio and ground into coarse powder. The powdered mixture was soaked in 10 volumes of distilled water for 60 min, then decocted by bringing to a vigorous boil followed by 40 min of sustained simmering. After cooling to ambient temperature, the decoction was filtered through an 8-layer sterile gauze to collect the primary extract. A secondary extraction was performed with 8 volumes of distilled water, repeating the identical protocol (Deng et al., 2022). The filtrates obtained from the two extraction steps were combined and concentrated under reduced pressure using a rotary evaporator to yield the final extract (10:1, w/w; specific gravity: 1.09).

**FIGURE 1:**
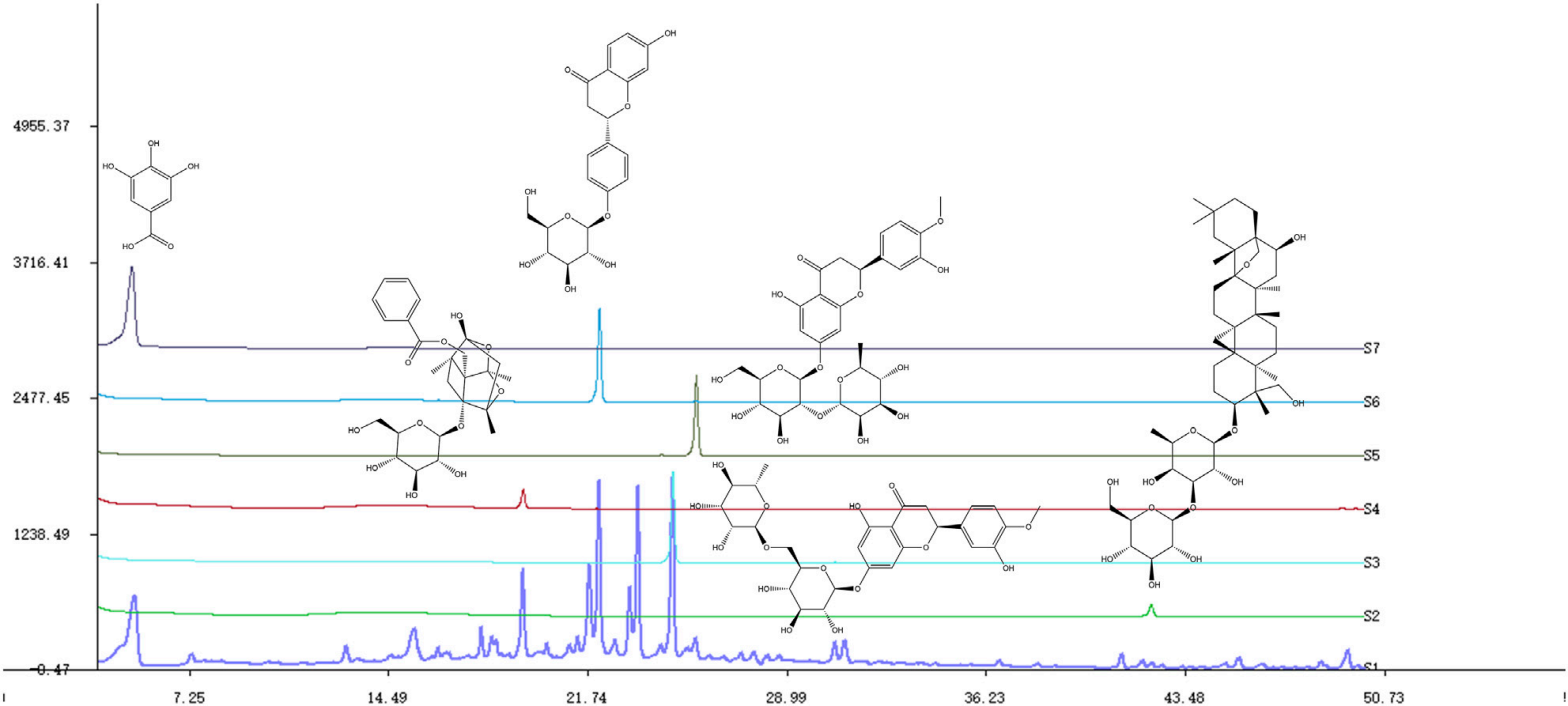
Chemical profiles of SNS and some compounds based on HPLC. S1-S7: SNS, saikosaponin A, hesperidin, paeoniflorin, neohesperidin, liquiritin, gallic acid.

The SNS extract was dissolved in methanol using ultrasonication to prepare a solution with a concentration of 1.2%. Saikosaponin A (A14GB145174), paeoniflorin (M28GB143089), liquiritin (Z07J12X136344), gallic acid (C17D10C105977), hesperidin (K09S11L123847) and Neohesperidin (G10S11L123540) from Shanghai Yuanye Bio-Technology Co., Ltd. (China) were weighed, dissolved in methanol, and moved to a 1 mL volumetric flask separately. SNS samples were analyzed using an Agilent HPLC-1290 System. A Diamonsil C18(2) (150 × 4.6 mm, 5 μm) was used to elute the analytes at 25°C. Samples were separated with a mobile phase consisting of A (acetonitrile) and B (0.01 mol/L aqueous phosphoric acid) solutions. The solvent gradients were as follows: 0–10 min with 2%–10% A; 10–20 min with 10%–22% A; 20–28 min with 22%–29% A; 28–40 min with 29%–40% A; 40–50 min with 40%–55% A. Of all the tests, the flow rate was set at 1 mL/min and the injection volume was 10 μL (Deng et al., 2022) (Figure 1).

### 2.2 Animal model

SD pregnant rats (14 days of gestation) were obtained from Charles River (Beijing, China). The animal model was prepared by maternal separation plus chronic unpredictable mild stress (MS-CUMS) (Huang et al., 2021). Starting from postnatal day 0 (PND0), male pups were individually separated from their dams during two daily sessions (9:00–12:00 and 14:00–17:00) and placed in clean containers lined with a small amount of home-cage bedding and cotton to maintain body temperature. After each separation session, pups were returned to their home cages for nursing. Control group pups remained undisturbed with their dams throughout this period. The MS procedure continued until weaning (PND21) (Cao et al., 2019). After weaning, pups were housed separately from dams for 1 week before initiating a secondary CUMS protocol at PND28. The CUMS regimen consisted of 32 days of varied stressors applied randomly (with no consecutive repeats), including: 24-h food deprivation, 24-h water deprivation, 24-h light/dark cycle reversal, 24-h cage crowding (5 rats/cage), 24-h exposure to wet bedding, 24-h cage tilt (45°), 5-min forced swim in warm water (45°C), 5-min cold exposure (5°C), 24-h empty water bottle exposure (Shen et al., 2020). Animals experiments were performed according to the guidelines of the National Institutes of Health Guide for the Care and Use of Laboratory Animals in Research and were approved by the Committee of Animal Experiment Ethics Review in Guangzhou University of Chinese Medicine (approval numbers: 22221110).

### 2.3 Grouping and drug administration

Male neonatal animals were randomized into 9 groups: Control, Model, Fluoxetine (FXT), SNS low/medium/high-dose (SNS-L/M/H), Control + sham-operation, Model + sham-operation, and Model + GdCl_3_ (30 μmol) with 8 pups in each group. From postnatal day 28 (PND28), drug intervention was administered to treatment group rats 2 h prior to daily CUMS exposure. Following body weight measurement, all rats received oral gavage at a standardized volume of 1 mL/100 g body weight. The FXT group and SNS-L/M/H groups were subjected to the MS-CUMS model and received daily oral administration of 5 mg/kg fluoxetine or 0.25/0.5/1 g/kg SNS extract, respectively, for 4 consecutive weeks. Model and Control groups received equivalent volumes of ultrapure water. After 3 weeks of modelling in adolescent rats, we administered GdCl_3_ by brain localization injection.

### 2.4 Cannula implantation and intra-DRN injections

Two weeks following CUMS induction, rats were anesthetized with 5% isoflurane (inhaled) and secured in a stereotaxic frame. A guide cannula was stereotactically implanted into the DRN (coordinates relative to bregma: AP -7.8 mm, ML -2.0 mm, DV -6.3 mm; 20° angle). Microinjections were administered at a constant rate of 0.2 μL/min using an infusion pump. Postoperative recovery was allowed for 7 days prior to further procedures.

### 2.5 Behavioral procedures

#### 2.5.1 Food intake and weight measurement

Throughout the 32-day experimental period, all rats received precisely measured feed quantities at weekly intervals. Residual feed was weighed at 08:00 each Monday using an analytical balance, with concurrent recording of body weights.

#### 2.5.2 Sucrose preference test (SPT)

Two identical water bottles fitted with pipette lids were prepared, each containing 1% (w/v) sucrose solution, and mounted on separate metal racks. After 24 h of habituation, one bottle was replaced with water, with their positions alternated at the 12 h midpoint to control for side preference. Following a 24 h deprivation period (food and water withheld), fluid consumption from both bottles was measured over a 24 h test session. Sucrose preference was calculated as: [sucrose intake (mL)/ (sucrose intake + water intake (mL))] × 100%, as previously validated (Meyerolbersleben, et al., 2020).

#### 2.5.3 Open field test (OFT)

At PND58, behavioral parameters were recorded for 5 min beginning 30 min post-drug administration using an automated video-tracking system. Quantified measures included total distance, central region distance and central region time for each subject.

#### 2.5.4 Forced swimming test (FST)

Animals were subjected to a 6-min forced swim test in a cylindrical chamber (25°C ± 1°C), with immobility duration quantified using SuperFst high-throughput forced swimming anxiety depression experiment software.

### 2.6 Western blot (WB)

Total proteins were extracted from rat DRN tissues using RIPA lysis buffer supplemented with protease and phosphatase inhibitors. Protein concentrations were quantified using the bicinchoninic acid (BCA) assay according to the manufacturer’s instructions. WB experiments refer to (Cao et al., 2024). For immunoblotting, the following primary antibodies were employed: Neuroligin 1 (bs-21796R, 1:1000) from Bioss (Beijing, China); PSD95 (GB11277, 1:1000), GAP43 (GB11095, 1:1000), SYN (GB11553, 1:5000) and Spinophilin (GB111956, 1:500) from Servicebio (Wuhan, China); CaSR (AF6296, 1:500) and Tubulin (AF7011, 1:5000) from Affinity (Jiangsu, China); GAPDH (MB66349, 1:5000) from Bioworld (Bloomington, United States); Goat anti-Rabbit IgG Secondary Antibody HRP conjugated (L3012, 1:5000) from Signalway Antibody (Maryland, United States). The ECL assay kit (PE0010, Solarbio, China) is used to visualize the signal.

### 2.7 Transmission electron microscope (TEM) study

Following the completion of behavioral assessments, rats were euthanized via intraperitoneal injection of sodium pentobarbital (50 mg/kg). The DRN was meticulously dissected and immersed in electron microscope fixative at 4°C for initial fixation. The pre-fixed DRN was cut into small pieces of 1 mm^3^, subsequently, the tissues were rinsed three times with 0.1 M phosphate-buffered saline (PBS). Post-rinsing, the tissues were subjected to secondary fixation using 1% osmium tetroxide at room temperature in the dark for 2 h, followed by an additional three PBS washes. Dehydration was performed using a graded ethanol series: 30%, 50%, 70%, 80%, 95%, and 100% ethanol. The 100% ethanol step was repeated twice to ensure complete dehydration. Tissues were further dehydrated twice in 100% acetone. After dehydration, the samples were placed in embedding molds and incubated overnight in a 37°C oven. The molds were then transferred to a 60°C oven for 48 h to facilitate resin polymerization. Ultrathin sections (60–80 nm) were obtained from the embedded resin blocks using an ultramicrotome (Leica UCT, Germany) and stained with 2% uranyl acetate in saturated ethanol solution for 8 min, followed by examination using a JEM1230 transmission electron microscope (Tokyo).

### 2.8 Hematoxylin-Eosin (HE) staining

Following behavioral testing, rat brains were perfusion-fixed with 4% paraformaldehyde (PFA) and post-fixed for 24 h. The fixed tissues were dehydrated, cleared, and embedded in molten paraffin. After solidification, the paraffin blocks were sectioned coronally at 4 μm thickness using a microtome. Sections were floated in a 47°C–48°C water bath, mounted on glass slides, and dried at 45°C. Hematoxylin and eosin (HE) staining was performed according to the manufacturer’s protocol (Beyotime, China). Histological analysis was conducted using bright-field microscopy.

### 2.9 Nissl staining

Sections were dewaxed and dehydrated, followed by washe with distilled water. Nissl staining was performed by incubating the sections in Nissl staining solution (Beyotime, China) at 37°C for 10 min. After staining, the sections were briefly rinsed twice with distilled water. Dehydration was conducted by immersing the sections in 95% ethanol twice. Subsequently, the sections were cleared in xylene twice for 5 min each to achieve transparency. Finally, the sections were mounted with neutral balsam and observed under a light microscope. Neuronal cell quantification was performed using ImageJ software (National Institutes of Health, United States).

### 2.10 Statistical analysis

Statistical analyses were performed using SPSS Version 21. Data were presented as mean ± SEM. One-way ANOVA test was employed for the purpose of comparing each experimental group. For *post hoc* analysis, Fisher’s least significant difference (LSD) test was applied when homogeneity of variance was confirmed. In cases where variances were heterogeneous, Dunnett’s T3 test was utilized for multiple comparisons. Pearson’s correlation coefficient was calculated to assess linear relationships between variables. p < 0.05 was considered statistically significant.

## 3 Results

### 3.1 SNS improves depressive-like behavior in the MS-CUMS model

**FIGURE 2:**
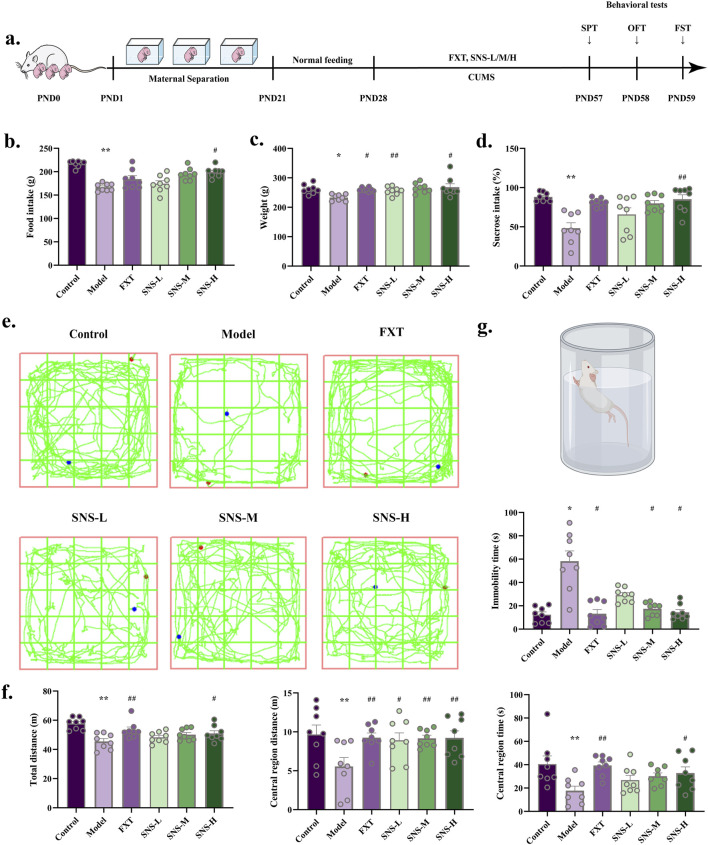
Antidepressant and anxiolytic effects of SNS. **(a)** MS-CUMS experimental procedures and behavioral tests. **(b)** Rat feeding (n = 8). **(c)** Rat body weight (n = 8). **(d)** Sucrose preference (%) in SPT (n = 8). **(e)** Sample travel paths in OFT. **(f)** OFT: distance and time (n = 8). **(g)** FST: immobility time (n = 8). *P < 0.05, **P < 0.01 VS. Control; #*P* < 0.05, ##*P* < 0.01 VS. Model.

The impact of SNS on behavioural performance were observed in MS-CUMS rat model (Figure 2A). Changes in intake during SNS intervention at various time periods showed that high doses of SNS, significantly attenuated the MS-CUMS-induced reduction in intake (*P* < 0.05). See Figure 2B. The Model group exhibited a considerable reduction in weight, which was significantly counteracted in SNS groups (*P* < 0.01, *P* < 0.05) (Figure 2C). A significant reduction in sucrose preference was observed in model group in comparison to control group (*P* < 0.01), yet it was reversed in the SNS-M groups (*P* < 0.01) (Figure 2D). Furthermore, total/central area distance and time were reduced in Model group (*P* < 0.01), yet reversed in FXT and SNS groups (*P* < 0.01, *P* < 0.05, Figures 2E,F). Antidepressant effects of both FXT and SNS were significant in FST (*P* < 0.05) (Figure 2G). This implies that MS-CUMS induces depression in rats, while FXT and SNS improved their depression-like behaviour. In summary, it is suggested that SNS has some antidepressant effect after application.

### 3.2 SNS ameliorates synaptic damage in DRN of model rats

**FIGURE 3:**
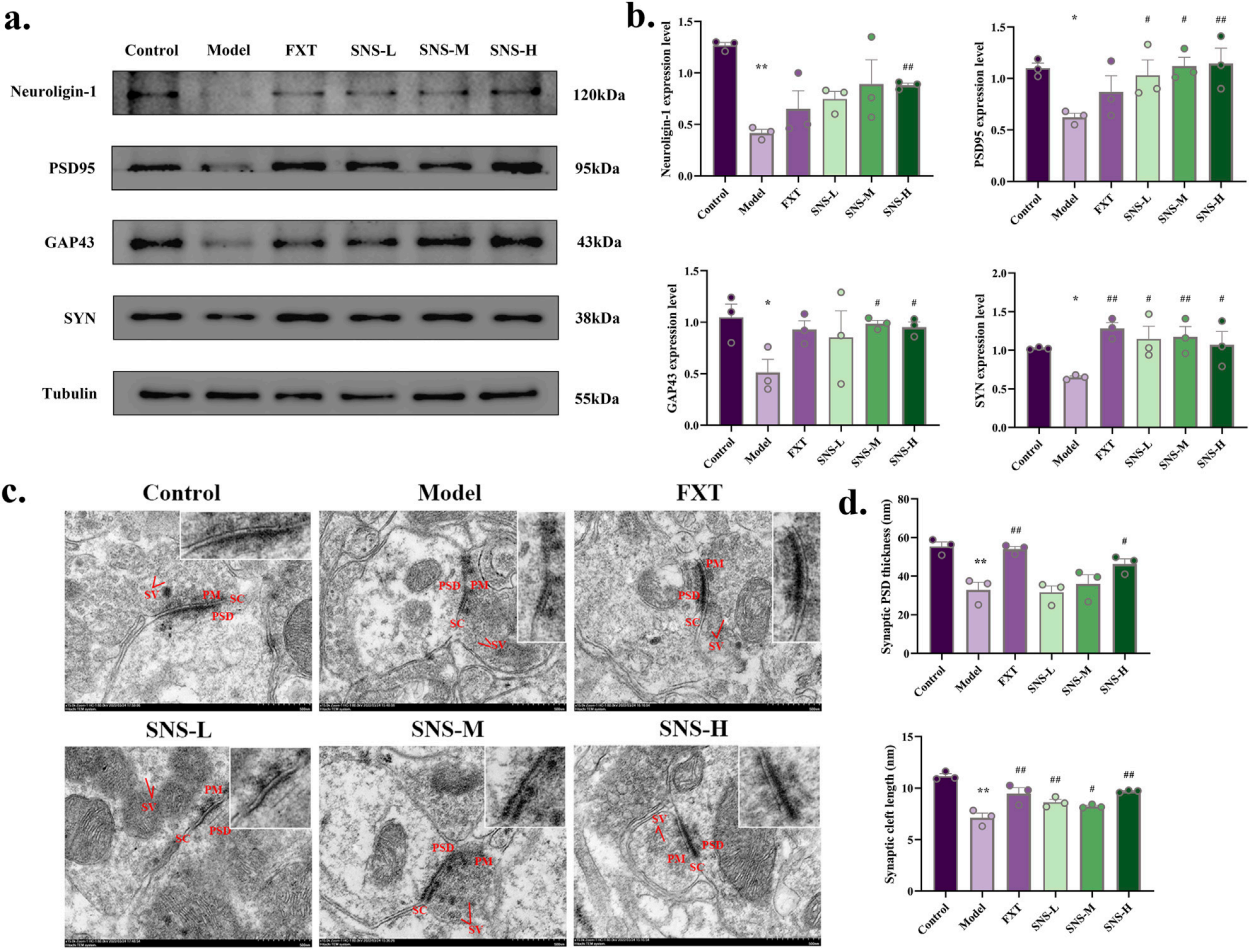
SNS upregulates synaptic-related proteins and ameliorates structural synaptic damage in the DRN of model rats. **(a)** Representative band plots of synaptic plasticity proteins in DRN. **(b)** Relative content of synaptic plasticity protein expression levels (n = 3). **(c)** Transmission electron microscopy samples of synaptic structures (scale bars of 500 nm). **(d)** Synaptic PSD thickness and synaptic cleft length (n = 3). *P < 0.05, **P < 0.01 VS. Control; #*P* < 0.05, ##*P* < 0.01 VS. Model. SV: synaptic vesicle; PSD: postsynaptic density; PM: presynaptic membrane; SC: synaptic cleft.

To substantiate the effect of SNS in improving synapses, WB was performed to observe the expression of neuroligin-1, PSD95, GAP43 and SYN proteins in DRN. In comparison to Control group, the expression of neuroligin-1, PSD95, GAP43 and SYN protein was significantly reduced in DRN of Model group (*P* < 0.01, *P* < 0.05). In contrast, SNS-H markedly enhance above mentioned proteins levels in DRN of model rats (*P* < 0.01, *P* < 0.05, Figures 3A,B). The synaptic postsynaptic density (PSD) thickness and cleft length (Figures 3C,D) were reduced in Model group, and reversed in FXT and SNS groups. These results demonstrate that SNS alleviates depressive-like behaviors by ameliorating synaptic structural damage in the DRN.

### 3.3 SNS improves the pathological structure and CaSR expression of DRN in early life stress-depressed rats

**FIGURE 4:**
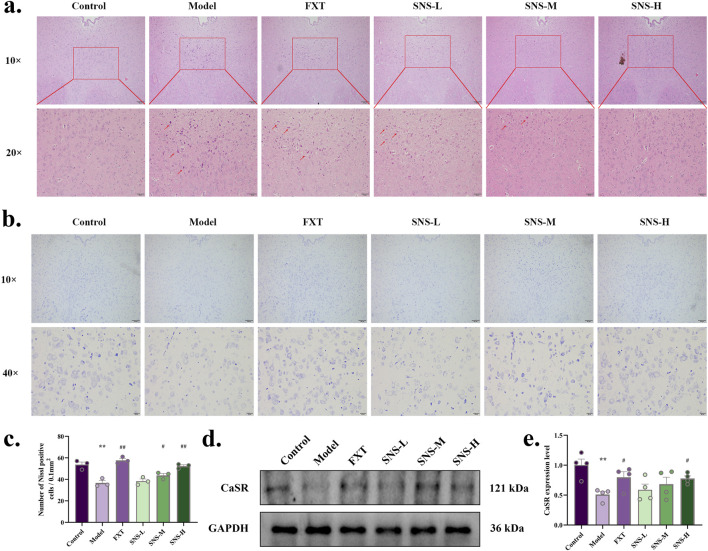
SNS improves pathological structure and CaSR expression level of DRN in early life stress-depressed rats. **(a)** HE staining. The scale is 100 and 20 μm. **(b)** Nissl staining. The scale is 100 and 20 μm. **(c)** Comparison of the number of DRN nissl body in each group of rats. **(d)** Representative band plots of CaSR protein in DRN. **(e)** Relative content of CaSR protein expression level. *P < 0.05, **P < 0.01 VS. Control; #*P* < 0.05, ##*P* < 0.01 VS. Model.

The neurons in DRN of Model rats underwent significant degeneration, resulting in a reduction in their size (Figure 4A). In comparison to Model group, all treated groups showed some degree of improvement. The results of nissl staining of DRN are presented in Figures 4B,C. The neurons of model rats were sparsely arranged and the outline of the cell body was not obvious. Nevertheless, the histopathological manifestations of model rats DRN were improved in FXT and SNS-M/H groups, with significantly increased numbers of treated nissl bodies (*P* < 0.01, *P* < 0.05). To further confirm the impact of SNS on alterations in CaSR, WB was conducted to observe level of CaSR protein in DRN. The results are shown in Figures 4D,E. MS-CUMS significantly downregulated CaSR protein expression in DRN tissues (*P* < 0.01). In comparison to model group, the protein expression was markedly upregulated in DRN tissues of rats in FXT and SNS-H group (*P* < 0.05).

### 3.4 CaSR is involved in adolescent depression

**FIGURE 5:**
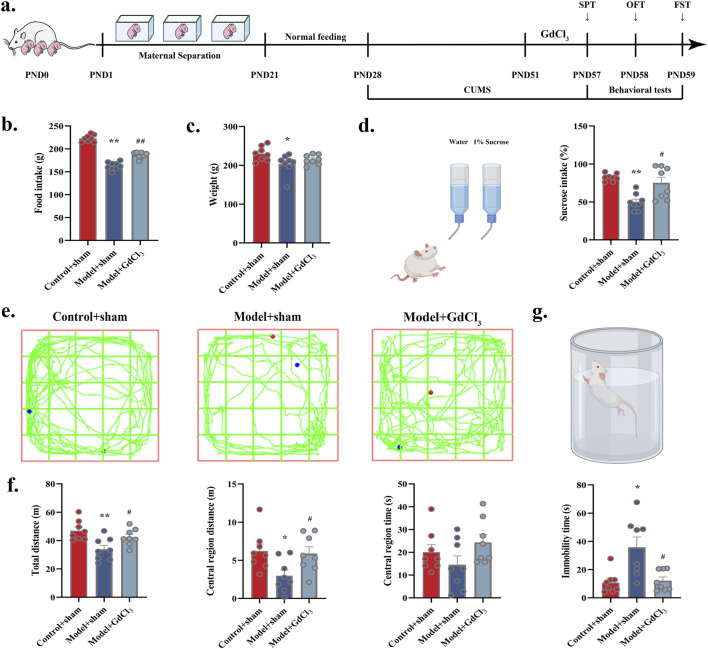
Administration of CaSR agonist (GdCl_3_) improved depression-like behaviour of adolescent rats: **(a)** Experimental flow. **(b)** Food intake in rats. **(c)** Body weight in rats. **(d)** Diagram of the trajectory of the manoeuvre in the OFT. **(e)** Sucrose intake (%). **(f)** Total distance, central region distance and time. **(g)** Immobility time in FST. *P < 0.05, **P < 0.01 VS. Control + sham; #*P* < 0.05, ##*P* < 0.01 VS. Model + sham.

To determine the contribution of CaSR in pathogenesis of depression, individual behavioral tests were preceded by treatment with CaSR agonist (GdCl_3_), and then depressed behavior was observed in control and stress-exposed rats. At the end of modelling, administration of the CaSR agonist GdCl_3_ significantly increase food intake (*P* < 0.01) as well as sucrose preference (*P* < 0.05) and significantly improved spontaneous activity in model rats (total distance: *P* < 0.05; central region distance: *P* < 0.05). Furthermore, immobility time was significantly shorter in model + GdCl_3_ group (*P* < 0.05). The findings indicate that CaSR may indeed play a role in depressive pathogenesis (Figure 5).

### 3.5 GdCl_3_ ameliorates synaptic structural impairments in the DRN of adolescent depressive rats

**FIGURE 6:**
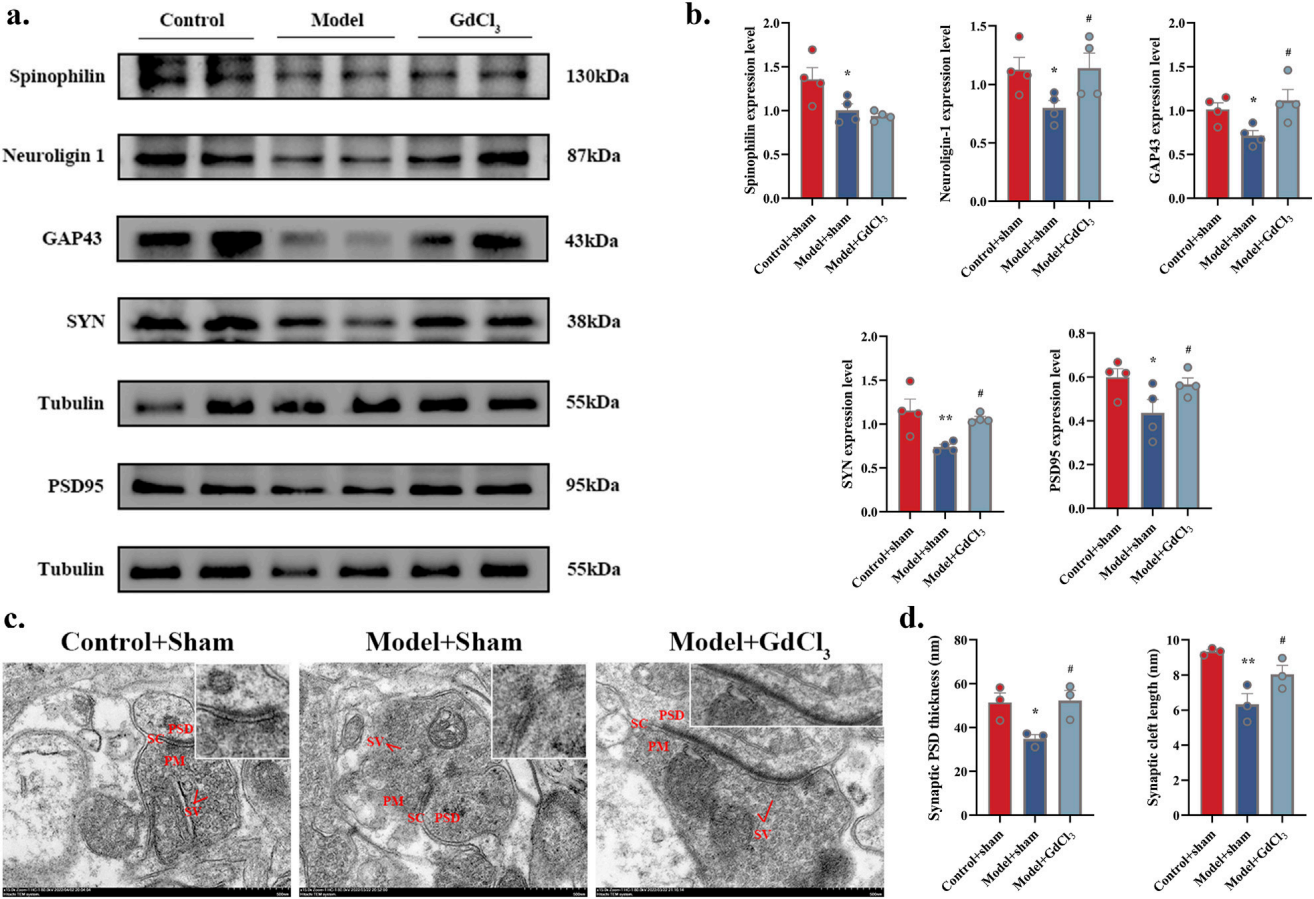
Administration of the CaSR agonist GdCl_3_ ameliorates synaptic structural damage in adolescent depressive rats. **(a,b)** Neuroligin-1, GAP43, SYN, and PSD95 protein levels (n = 4 rats per group). **(c)** Sample synaptic structures (scale bar of 500 nm). **(d)** Synaptic PSD thickness and synaptic cleft length (n = 3). *P < 0.05, **P < 0.01 VS. Control + sham; #*P* < 0.05 VS. Model + sham. SV: synaptic vesicle; PSD: postsynaptic density; PM: presynaptic membrane; SC: synaptic cleft.

WB was performed to observe the expression of spinophilin, neuroligin-1, GAP43, SYN, and PSD95 proteins in DRN. Significant increases in the expression of neuroligin-1, GAP43, SYN, and PSD95 were observed in the DRN treated with GdCl_3_ (*P* < 0.05, Figures 6A,B). The synaptic PSD thickness and cleft length (Figures 6C,D) were reduced in Model + Sham group, and reversed in Model + GdCl_3_ group. These results demonstrate that GdCl_3_ alleviates depressive-like behaviors by ameliorating synaptic structural damage in the DRN.

### 3.6 Correlation of DRN CaSR and synaptic-related protein expression levels with depressive behavior

**FIGURE 7:**
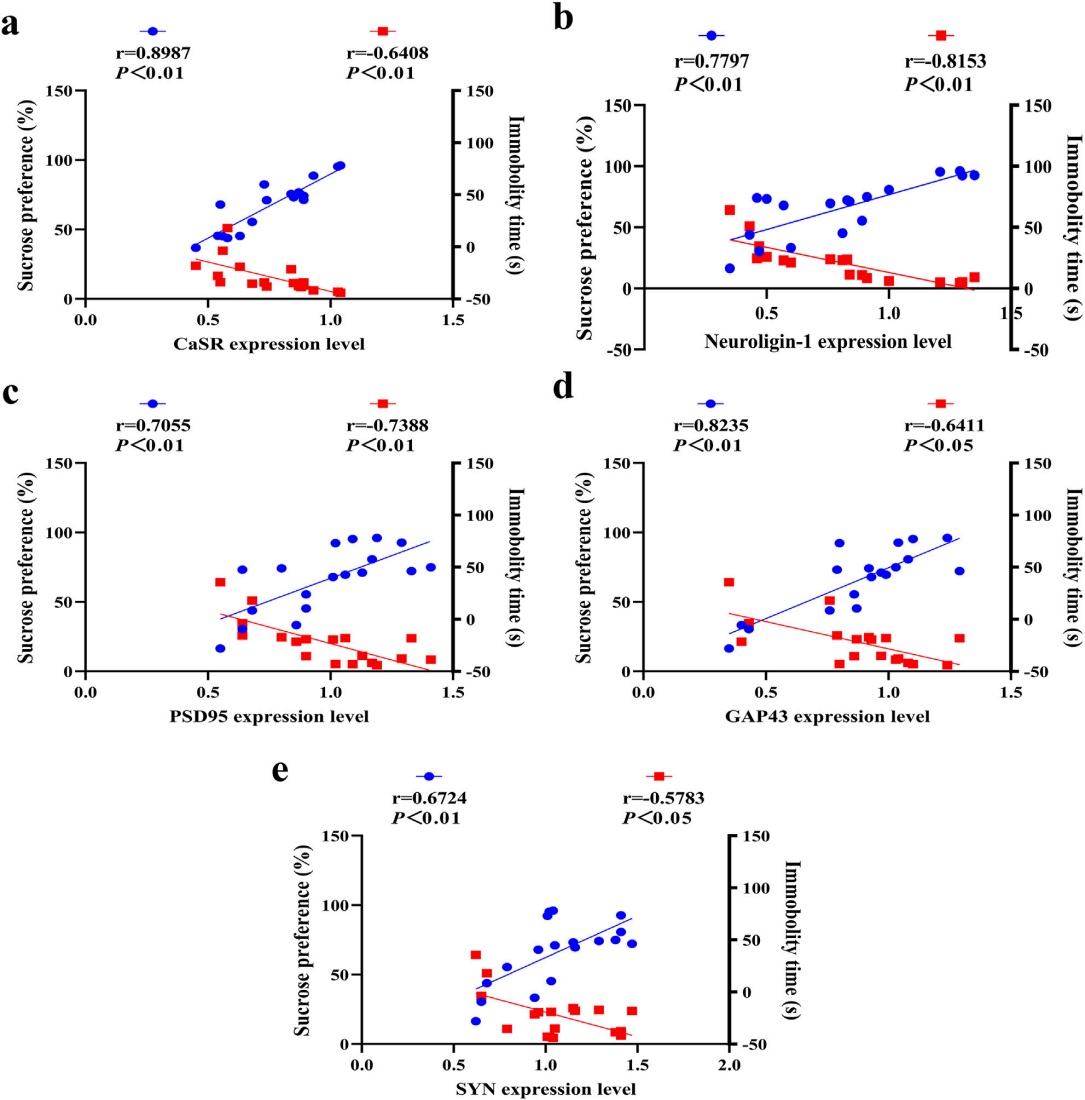
Correlations between CaSR/synaptic protein expression in the DRN and depressive behaviors. Correlation of depression-like behavior tests with **(a)** CaSR, **(b)** neuroligin-1, **(c)** PSD95, **(d)** GAP43 and **(e)** SYN expression levels. The blue data points represent correlation analyses between protein expression levels and sucrose preference (%), while the red data points indicate correlation analyses between protein expression levels and immobility time (s).

A linear regression analysis was performed using the behavioral outcome of adolescent depressed rats as the dependent variable and the expression levels of CaSR, neuroligin-1, PSD95, GAP43 and SYN as the independent variables. The study revealed significant correlations between sucrose preference (%)/immobility time and expression levels of CaSR (Figure 7A), neuroligin-1 (Figure 7B), PSD95 (Figure 7C), GAP43 (Figure 7D), and SYN (Figure 7E). In summary, the lower CaSR expression level,the more severe the impairment in synaptic plasticity, the more pronounced the depressive mood in adolescent depressed rats. By improving CaSR and synaptic plasticity, SNS alleviates depressive-like behavior.

## 4 Discussion

The results showed the efficacy of MS-CUMS in inducing depressive-like behavior in adolescent rats. In addition, MS-CUMS impaired synaptic ultrastructure in the DRN while decreasing the expression of synapse-associated proteins. Moreover, MS-CUMS decreased CaSR activity in the DRN, and increased CaSR expression in DRN was found to reverse depression-related behaviours and lead to synaptic structural resonance and increased expression of synapse-associated proteins, suggesting that CaSR is a crucial mediator of MS-CUMS-induced behaviours and synaptic abnormalities. In addition, SNS was observed to mitigate depressive-like behaviour in adolescent rats, while also reversing MS-CUMS-induced synaptic abnormalities in the DRN. Furthermore, SNS increased CaSR expression in the DRN. These findings demonstrate that SNS may exert antidepressant effects by upregulating CaSR expression in the DRN, thereby ameliorating synaptic structural impairments.

To assess the antidepressant effect, intake, body weight, SPT, FST and OFT were performed. Food intake is a valid indicator of depressed mood. The present experiment revealed that after MS-CUMS modeling in adolescent rats, compared with the control group, there was a notable reduction in food intake. The reduced food intake in the rats after modeling was very similar to the clinical manifestations of depression, and the findings align with previous reports. There was also a notable reduction in body weight after MS-CUMS. Body weight measurements were performed to examine the eating habits and metabolic status of the rats, as the model rats showed a decrease in diet and subsequent weight loss. Food intake and body weight increased substantially after administration of the CaSR agonist GdCl_3_. Additionally, similar to other antidepressants (Serretti and Mandelli, 2010), SNS demonstrated a significant reversal of reduction in appetite and body weight in depressed rats. Sucrose intake was significantly lower in Model rats, indicating that MS co-CUMS resulted in a lack of pleasure and decreased arousal. FST immobility time was markedly elevated in model rats, suggesting MS-CUMS-induced desperate behavior. Total OFT distance, central area distance and time were significantly lower in model rats, indicating anxiety-like behavioral changes in MS-CUMS rats. The above behavioral results are consistent with a number of other models of early life depression (Amini-Khoei et al., 2017). Behavioural experiments have shown that GdCl3 is more effective in alleviating depressive-like behaviours. This suggests that SNS can reverse behavioral abnormalities caused by MS-CUMS, and that this process may be related to CaSR. Despite the behavioral confirmation, the pharmacological mechanism remains unknown.

The DRN plays pivotal role in stress response and abnormal behavior. Previous study suggests that early life stress may induce changes in persistent neuronal activity in the DRN, which may underlie an individual’s increased sensitivity to stress (Lukkes et al., 2009). Previous research has demonstrated that early life stress impairs synaptic plasticity and may lead to emotional dysfunction in adulthood (Herpfer et al., 2012). The mechanisms by which early-life stress alters synaptic structure and function in the adolescent DRN remain incompletely understood. Our findings indicate that MS-CUMS impaired synaptic ultrastructure in male mice. In addition, MS-CUMS reduced expression levels of synapse-associated proteins including neuroligin-1, PSD95, GAP43 and SYN. Our findings corroborate previous research showing that stress exposure impairs synaptic plasticity in DRN of adolescent individuals (Sun et al., 2024). Similarly, repeated restraint stress rats have been reported to exhibit an increased frequency of spontaneous EPSC in DRN (Bąk et al., 2022). As previously demonstrated by our research (Zhou et al., 2022), DRN neuronal alterations in mice during tail-hanging experiments were associated with despair-like behavior. This study is one of a limited number of studies that assess the effects of early-life stress on synapses in the adolescent dorsal raphe nucleus (DRN). Many proteins are involved in the process of synaptic remodeling, such as GAP43 and SYN (Chato-Astrain et al., 2023). Previous study has similarly reported that social isolation significantly reduced PSD95 and SYN protein expression in the DRN region (Lee et al., 2021). Furthermore, synaptic plasticity impairments are associated with reduced neuronal complexity (Li et al., 2022). Therefore, we hypothesize that synaptic impairments in the DRN may indicate increased vulnerability to early-life stress. Recent finding showed that teenagers with MDD exhibit abnormal alterations in DRN dynamic functional connectivity, which is significantly associated with depressive symptoms during early life (Chen et al., 2021). Moreover, synapses are critically involved in functional brain connectivity. Collectively, these findings demonstrate that DRN synaptic alterations may constitute a pivotal factor in adolescent depression pathogenesis.

CaSR not only plays pivotal role in monitoring the maintenance of Ca^2+^ homeostasis, but also regulates axonal growth and differentiation of neurons during development (Breitwieser, 2006). Over time, there is evidence that CaSR plays a significant role in a variety of psychiatric diseases, including Alzheimer’s disease and depression (Shen et al., 2020). However, the role of CaSR has yet to be demonstrated in adolescent depressive-like behaviors. The findings suggest that MS-CUMS reduces CaSR expression, whereas activation of CaSR improves depressive-like behavior and prevents early-life stress-induced impairment of DRN synaptic. This is in accordance with our previous research indicating that CaSR plays a role in MS-CUMS-induced behavioral abnormalities and synaptic abnormalities in the hippocampus as well as prefrontal cortex (Shen et al., 2020). Furthermore, it is noteworthy that previous evidence from lipopolysaccharide mouse models suggests that phenol glycosides exert antidepressant effects by modulating CaSR level of hypothalamus (Feng et al., 2020). These findings further demonstrate the critical role of CaSR in synaptic regulation and antidepressant efficacy.

SNS is known for their ability to relieve depression and clear the liver and spleen, and this herbal combination is a classic formula for the treatment of depression. The antidepressant effect of SNS has been demonstrated in pharmacological experiments to be primarily attributed to regulation of 5-HT content, reduction of inflammatory immune response, and upregulation of neurotrophic factors (Cao et al., 2019; Li et al., 2013a; Yi et al., 2013). The multiple active constituents of SNS have been demonstrated to exert antidepressant effects through distinct pharmacological mechanisms. Saikosaponin A exhibits therapeutic potential by upregulating PRRT2 expression in the hippocampus while enhancing dopaminergic (DA) neurotransmission (Guo et al., 2020). Hesperidin mediates its effects through activation of the Nrf2/ARE/Glyoxalase 1 pathway, particularly in streptozotocin-induced diabetic depression models (Zhu et al., 2020). Paeoniflorin orchestrates comprehensive metabolic regulation via the citrate cycle and related pathways, effectively counteracting metabolic disturbances in CUMS-induced depression (Lei et al., 2022). Neohesperidin demonstrates unique efficacy in reversing prednisolone-induced depressive behaviors through selective activation of mTORC1 signaling (Deyama et al., 2024). Liquiritin modulates the neuro-endocrine-immune network with particular relevance to menopausal depression management (Lan et al., 2020). Gallic acid emerges as a multitarget agent capable of alleviating depression-pain comorbidity through selective inhibition of P2X7 receptor expression across hippocampal, spinal cord, and dorsal root ganglion tissues (Wen et al., 2022).

This study have observed that SNS intervention yields promising therapeutic outcomes for depression-like behaviour. The effect of SNS to improve synaptic damage in hippocampus and prefrontal cortex was reported in our previous study (Cui et al., 2020). But what is SNS affects synaptic structure of DRN is not yet known. We therefore observed rat DRN synaptic morphology and function by TEM, excitingly, the SNS had an impact on improving synaptic damage. In addition, SNS reversed the reduced expression of PSD95, SYN, neuroligin-1 and GAP43 proteins in the DRN brain region MS co-CUMS model, in line with previous depression models (McEwen et al., 2012; Liu and Aghajanian, 2008) and some antidepressant research (Li et al., 2010; Voleti et al., 2013). To elucidate the precise mechanism underlying SNS-mediated synaptic repair, we focused on investigating CaSR regulation. CaSR expression results showed a decrease in the MS-CUMS model, and was reversed by SNS intervention. To clarify whether depression-like behavior and CaSR are correlated, we analyzed the correlation between CaSR levels and depression-like behaviors. CaSR was significantly associated with depressive-like behavior. Collectively, SNS ameliorates synaptic structural impairments in the DRN of adolescent depressive rats, potentially through upregulated CaSR protein expression. It was shown that intracellular Ca^2+^ overload-triggered waterfall-like chain reaction is a common pathway for neuronal injury, and CaSR has the function of sensing changes in Ca^2+^ concentration and regulating neighboring neurons (Spitzer, 2008). High Ca^2+^ activates CaSR and thus promotes axonal growth of sympathetic neurons in late mouse embryos. Recent studies have also found that CaSR expression on neurons is involved in the control of Na leakage channels and that the activation of this control is based on a reduction in extracellular Ca^2+^ levels (Phillips et al., 2008). Specific bioactive components of Sinisan (SNS) exhibit neuroprotective effects through calcium homeostasis regulation, as evidenced by saikosaponins demonstrating significant protection against corticosterone-induced apoptosis in PC12 cells via modulation of Ca^2+^ equilibrium (Li et al., 2013b). Similarly, paeoniflorin effectively counteracts NMDA-triggered intracellular Ca^2+^ overload in PC12 cells while restoring physiological levels of calbindin-D28k mRNA expression (Mao et al., 2011). These findings collectively underscore the pharmacodynamic synergy of SNS constituents in maintaining neuronal calcium signaling integrity under pathological conditions. We therefore hypothesized that SNS may multicomponentally regulate Ca^2+^ levels and thereby activate CaSR to exert antidepressant effects. However, our study has not delved into the role of active ingredients in SNS in MS-CUMS-induced depressive-like behaviors. Further research is required to explore different therapeutic components and specific mechanisms underlying their effects on CaSR activity.

## 5 Conclusion

Collectively, our findings substantiate the pivotal role of CaSR in mediating MS-CUMS-induced behavioral abnormalities during early-life stress. Moreover, we demonstrate that SNS exerts antidepressant effects by restoring CaSR expression and ameliorating synaptic deficits in the DRN. Further investigations are warranted to fully elucidate the precise molecular mechanisms underlying these therapeutic effects.

## Data Availability

The raw data supporting the conclusions of this article will be made available by the authors, without undue reservation.
